# Impact of Early Pandemic SARS-CoV-2 Lineages Replacement with the Variant of Concern P.1 (Gamma) in Western Bahia, Brazil

**DOI:** 10.3390/v14102314

**Published:** 2022-10-21

**Authors:** Josilene R. Pinheiro, Esther C. dos Reis, Jéssica P. Farias, Mayanna M. C. Fogaça, Patrícia de S. da Silva, Itana Vivian R. Santana, Ana Luiza S. Rocha, Paloma O. Vidal, Rafael da C. Simões, Wilson B. Luiz, Alexander Birbrair, Renato S. de Aguiar, Renan P. de Souza, Vasco A. de C. Azevedo, Gepoliano Chaves, Aline Belmok, Ricardo Durães-Carvalho, Fernando L. Melo, Bergmann M. Ribeiro, Jaime Henrique Amorim

**Affiliations:** 1 Center of Biological Sciences and Health, Federal University of Western Bahia, Barreiras 47805, BA, Brazil; 2 Department of Biological Sciences, State University of Santa Cruz, Ilhéus 45662, BA, Brazil; 3 Department of Dermatology, School of Medicine and Public Health, University of Wisconsin-Madison, Madison, WI 53706, USA; 4 Department of Pathology, Federal University of Minas Gerais, Belo Horizonte 31270, MG, Brazil; 5 Department of Radiology, Columbia University Medical Center, New York, NY 10032, USA; 6 Department of Genetics, Ecology and Evolution, Federal University of Minas Gerais, Belo Horizonte 31270, MG, Brazil; 7 D’Or Institute of Research, Rio de Janeiro 22281, RJ, Brazil; 8 Department of Pediatrics, University of Chicago, Chicago, IL 60637, USA; 9 Laboratory of Baculoviruses, University of Brasilia, Brasilia 70910, DF, Brazil; 10 Department of Microbiology, Immunology and Parasitology, São Paulo School of Medicine, Federal University of São Paulo (UNIFESP), São Paulo 04023, SP, Brazil; 11 Post-Graduate Program in Structural and Functional Biology, UNIFESP, São Paulo 04023, SP, Brazil

**Keywords:** COVID-19, impact, variant of concern

## Abstract

Background: The correct understanding of the epidemiological dynamics of COVID-19, caused by the SARS-CoV-2, is essential for formulating public policies of disease containment. Methods: In this study, we constructed a picture of the epidemiological dynamics of COVID-19 in a Brazilian population of almost 17000 patients in 15 months. We specifically studied the fluctuations of COVID-19 cases and deaths due to COVID-19 over time according to host gender, age, viral load, and genetic variants. Results: As the main results, we observed that the numbers of COVID-19 cases and deaths due to COVID-19 fluctuated over time and that men were the most affected by deaths, as well as those of 60 or more years old. We also observed that individuals between 30- and 44-years old were the most affected by COVID-19 cases. In addition, the viral loads in the patients’ nasopharynx were higher in the early symptomatic period. We found that early pandemic SARS-CoV-2 lineages were replaced by the variant of concern (VOC) P.1 (Gamma) in the second half of the study period, which led to a significant increase in the number of deaths. Conclusions: The results presented in this study are helpful for future formulations of efficient public policies of COVID-19 containment.

## 1. Introduction

The *Severe Acute Respiratory Syndrome Coronavirus 2* (SARS-CoV-2) belongs to the Betacoronavirus genus in the Coronaviridae family [1]. It is the causative agent of the coronavirus disease 2019 (COVID-19) [2,3], the world’s major public health problem in the last three years, which affected hundreds of million people and caused more than 6 million deaths [4]. Due to the high epidemiological impact of the disease, a great effort was carried out by the scientific community around the world to rapidly study the virus and the disease. Relevant scientific progress was rapidly achieved regarding the knowledge of pathophysiology, transmission, diagnosis, and treatment [5,6,7,8,9,10]. In addition, several vaccine formulations were developed and are being shown as essential in the control of severe forms of COVID-19 worldwide [11,12]. However, the consecutive emergence of new genetic variants of SARS-CoV-2 have brought new questions and challenges to pathophysiology, transmission, diagnosis, treatment, and the use of vaccines [13].

The correct understanding of the epidemiological dynamics of COVID-19 is essential for formulating public policies of disease containment. For example, knowing when and why a new wave of COVID-19 is expected to happen can be used for preparedness in terms of the use of non-pharmacological measures, vaccination, reinforcement in the number of health professionals, reinforcements in laboratory tests availability, reinforcements in the numbers of hospital beds, and the cancellation of events, etc. [14,15,16]. Understanding the dynamics of COVID-19 cases and deaths over time, and according to gender and age, is also essential to predict situations of epidemiological risk and for preparedness regarding specific groups [17,18]. In addition, the knowledge about viral genetic variants circulating in a given area [19], their epidemiological impact and their probable origins, are also essential for successful public health policies of containment of importing or exporting viruses.

In this study, we aimed to have a picture of the epidemiological behavior of COVID-19 in a population study of almost 17,000 patients in 15 months. We specifically aimed to: (i) see the fluctuations of COVID-19 cases and deaths during the period of study; (ii) describe and understand the epidemiological behavior of COVID-19 according to patients gender, age, viral load, and viral genetic variants; (iii) to test possible associations between these variables; and (iv) to phylogenetically reconstruct the evolutionary relationships of SARS-CoV-2 circulating lineages sampled from the study. The results presented in this study are helpful for the future formulation of public policies of COVID-19 containment.

## 2. Materials and Methods

### 2.1. Data Collection

This is a retrospective study of the cases of COVID-19 registered in the cities of Western Bahia (west region of Bahia state, Brazil) from May 2020 to July 2021. All patient data and samples were provided by the Laboratory of Infectious Agents and Vectors from Western Bahia Federal University, located in Barreiras city, Bahia, Brazil. Information such as the patient’s name, age, gender, sample identification number, collection date, RT-qPCR result for SARS-CoV-2 detection with the value of the cycle threshold (Ct), the date of onset of symptoms, the municipality of residence, and the patient’s care unit, were used in this study. These data were tabulated and used in statistical analyses. All the research complied with all relevant ethical and biosafety guidelines. Ethics approval was obtained from the institutional ethics committee of the Federal University of Western Bahia (CAAE 40779420.6.0000.8060). All procedures and possible risks were explained to volunteers. Informed consent was obtained from all participants. The research was performed in accordance with relevant guidelines/regulations. The sample is composed of data from 16,908 laboratory tests, including positive and negative results of SARS-CoV-2 detection.

### 2.2. RNA Extraction and RT-qPCR

The nucleic acid extractions of nasopharyngeal samples were carried out using the Total RNA Purification Kit (Cellco Biotec, Sao Carlos, SP, Brazil), following the manufacturer’s protocol. We also carried out viral RNA extraction using the Extracta Kit—RNA e DNA Viral (MVXA-P016FAST) (Loccus, Sao Paulo, SP, Brazil), using an Extracta32 instrument (Loccus, Sao Paulo, SP, Brazil), following the manufacturer’s instructions.

Reverse transcription, followed by quantitative polymerase chain reaction (RT-qPCR) assays, were carried out as previously described by us [20]. Thermocycling was carried out in a QuantStudio 5 instrument (Applied Biosystems, Waltham, MA, USA) with a hold stage composed of a first step of 5 min at 50 °C, followed by a second step of 20 s at 95 °C. The PCR stage was composed of a first step of 15 s at 95 °C followed by a second step of 1 min at 55 °C, repeated 45 times. We also used Allplex 2019-nCOV RT-qPCR kit (Seegene, Song-pa-gu, Seoul, Republic of Korea), following the manufacturer’s instructions.

### 2.3. Viral Genotyping by RT-qPCR

Viral variants were characterized using RhAmp technology (Integrated DNA Technologies IDT, Coralville, IA, USA) and TaqMan SARS-CoV-2 Mutation Panel (Thermo Fisher, Waltham, MA, USA) with specific primers and probes targeting the VOCs defining mutations: K417T (A22812C), E484K (G23012A), and N501Y (A23063T). RhAmp and TaqMan detailed protocols were previously reported [21,22].

### 2.4. SARS-CoV-2 Genome SEQUENCING

One hundred and twenty positive samples collected from May 2020 to July 2021 were sequenced using Next Generation Sequencing (NGS) on the Oxford Nanopore’s MinIon platform. Viral RNA was extracted as described above. The RNA samples were submitted to reverse transcription with random primers using LunaScript® (New England Biolabs, Inc., Ipswich, MA, USA) or SuperScript® IV First-Strand Synthesis System (ThermoFisher Scientific, Waltham, MA, USA), as previously described (nCoV-2019 sequencing protocol v3 (LoCost) (protocols.io) [23]. The cDNA obtained was used as a template for the amplification of the entire genome of SARS-CoV-2 with the following primer scheme: a 400bp amplicon scheme from ARTIC nCoV-2019 sequencing protocol (v3) (nCoV-2019 sequencing protocol v3 (LoCost) (protocols.io) was used, as previously described [23]. End-prep reactions were performed with NEBNext® Ultra™ II End Repair/dA-Tailing Module, and amplicons were barcoded using the ONT Native Barcoding Expansion kit (EXP-NBD104). The barcoded samples were then combined, purified with AMPure XP Beads, and loaded onto Oxford Nanopore MinION SpotON Flow Cells R9.4.1 (Oxford Nanopore Technologies), following the manufacturer’s instructions. The sequencing was carried out using the fast accuracy base-calling in the MinKNOW software. ARTIC Network’s RAMPART (https://artic.network/ncov-2019, accessed on 2 December 2021) was used to monitor the sequencing run in real-time to estimate the depth of coverage (20×) across the entire genome for each barcode (https://artic.network/rampart, accessed on 2 December 2021). The analysis and consensus generation were performed according to the pipeline proposed by ARTIC Network using the Medaka protocol (https://artic.network/ncov-2019/ncov2019-bioinformatics-sop.html, accessed on 2 December 2021). All consensus genomes were deposited in the Global Initiative on Sharing Avian Influenza Data-EpiCoV (GISAID-EpiCoV) database (see Supplementary Material S1, for details).

### 2.5. Phylogenetic Analysis

New SARS-CoV-2 whole-genome sequences obtained here were submitted to lineages assigner Pangolin web application, available online: https://pangolin.cog-uk.io/, accessed on 1 September 2022. Initially, phylogenetic reconstructions were performed using datasets containing sequences from the study (n = 112) and 1004 representative Nextstrain’s subsampling SARS-CoV-2 genomic sequences from South America countries and their territories (n = 16). Such sequences were retrieved from the beginning of the pandemic until August 2022 (https://nextstrain.org/, accessed on 1 September 2022), representing multiple SARS-CoV-2 circulating strains. In addition, high-coverage complete SARS-CoV-2 Gamma genome sequences (n = 14846) from all Brazilian States and the Federal District (n = 27), deposited up to 31 July 2022 in the GISAID-EpiCoV, were also downloaded. Data sets were filtered out by the Sequence Cleaner, a biopython-based script (https://biopython.org/wiki/Sequence_Cleaner, accessed on 1 September 2022), which comprised a set of unambiguous sequences ≥ 29,000 bp with 0% of Ns and degenerated nucleotides. Sequences that did not fit these criteria were automatically excluded. The outcomes were aligned with the SARS-CoV-2 reference coding-sequence (NC_045512.2) by MAFFT v.7 [24] and edited by the UGENE v.44.0 [25]. 

Aiming to investigate the relative amount of unresolved to fully resolved trees, the phylogenetic signal approach was explored through likelihood mapping analysis of 10,000 random quartets using TREE-PUZZLE v.5.2 [26]. Then, the maximum likelihood (ML) method was implemented by using two different command line algorithms: FastTree v.2.1.7 [27] and IQ-TREE v.2 [26]. FastTree was executed by using the GTR substitution model + CAT with 20 gamma (G) distribution parameters and a mix of Nearest-Neighbor Interchanges (NNI) and Sub-Tree-Prune-Regraft (SPR). ML from IQ-TREE was inferred using the substitution model GTR + F + I + G4, executed and optimized by the Maximum Parsimony and Neighbor-Joining trees, and hill-climbing algorithms, respectively [28]. The reliability of the nodes was analyzed by the Shimodaira–Hasegawa (SH-like) test, which uses bootstrap resampling and corrects critical values for multiple comparisons [29], and SH-aLTR/aBayes/ultrafast bootstrap support values, both with 1000 replicates. Phylogenetic trees were generated by Interactive Tree of Life [30].

### 2.6. Statistical Analyses

To compare the means of two groups we used *t*-student test. To compare more than two groups we used analysis of variance (ANOVA) followed by Bonferroni multiple comparison test. In addition, to verify relations between variables we carried out linear regression analyses. In all cases, statistical significance was set as *p* ≤ 0.05. In some cases, we carried out descriptive statistics.

## 3. Results

### 3.1. Fluctuations of Numbers of COVID-19 Cases and Deaths from May 2020 to July 2021

**Figure 1:**
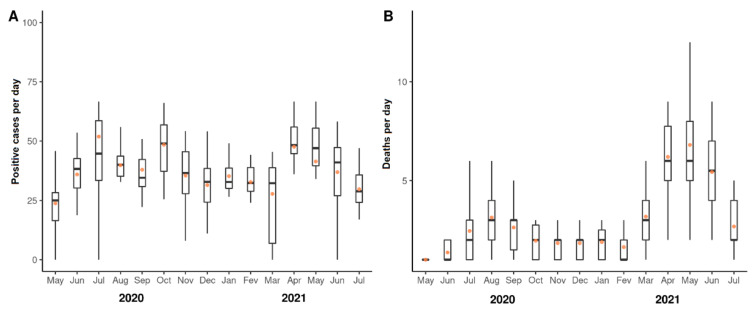
Fluctuations of COVID-19 cases and deaths due to COVID-19 during the period of study. (**A**) numbers of cases of COVID-19 per day during the period of study. (**B**) numbers of deaths due to COVID-19 per day during the period of study. Dots represent means. Horizontal bars represent medians.

The number of COVID-19 cases per day fluctuated from May 2020 to July 2021, with statistically significant peaks in August 2020, and in April and May 2021 (Figure 1A and Supplementary Table S1). The numbers of deaths per day due to COVID-19 also fluctuated in the study period (Figure 1B and Supplementary Table S2). First, relevant increases in the numbers of deaths per day were observed on July and October 2020. Then, significant increases were observed in April–June 2021. Collectively, these results indicate that the study population was relevantly affected by COVID-19, with different waves of cases and deaths over the time of study.

### 3.2. Impact of COVID-19 According to Gender or Age

**Figure 2:**
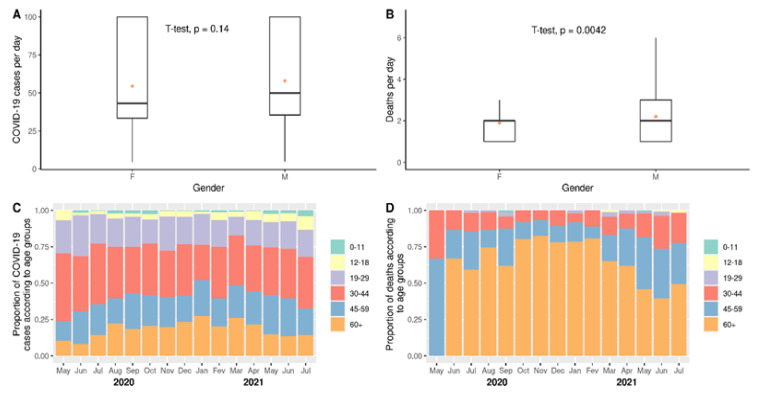
Impact of COVID-19 according to gender or age. Comparisons of numbers of cases per day (**A**) and numbers of deaths per day (**B**) considering the whole period of study were carried out based on Student’s *t*-test. Significance was set as *p* ≤ 0.05. In addition, proportions of cases (**C**) and deaths (**D**) according to age groups were computed. Dots represent means. Horizontal bars represent medians.

The numbers and rates of COVID-19 cases and deaths due to COVID-19 per day were also computed. As shown in Figure 2A, there was not a significant difference in the rates of cases according to gender. However, as shown in Figure 2B, the numbers of men dying of COVID-19 per day were significantly higher than those for women (*p* = 0.0042) during the study period. In addition, the proportions of cases and deaths in age groups 0–11, 12–18, 19–29, 30–44, 45–59, and 60 and above years old were observed. As shown in Figure 2C, the 30–44 years old age group was the most affected by COVID-19 cases during the study period (see Supplementary Table S3 for absolute numbers and descriptive statistics). This group was followed by those 45–59 and 60 and above years old in the proportions of COVID-19 cases.

In contrast, the group 60 and above years old, was the most affected by deaths due to COVID-19 in most of the period of study (Figure 2D and Supplementary Table S4 for absolute numbers and descriptive statistics). Such a leadership in the numbers of deaths was not observed only in the first month of the study and in May–July 2021, when the sum of numbers of deaths in the groups of 30–44 and 45–59 years old was higher than in the oldest group. It is essential to highlight that even in the three last months of the study, the oldest group was the most affected by deaths when compared pair-to-pair with the other age groups. Collectively, these results indicate that: (i) men were the most affected by deaths due to COVID-19; (ii) the age group of 30–44 years old was the most affected by COVID-19 cases; and (iii) the age group of 60 and above years old was the most affected by deaths due to COVID-19.

### 3.3. Viral Loads According to Cycle Threshold Values

**Figure 3:**
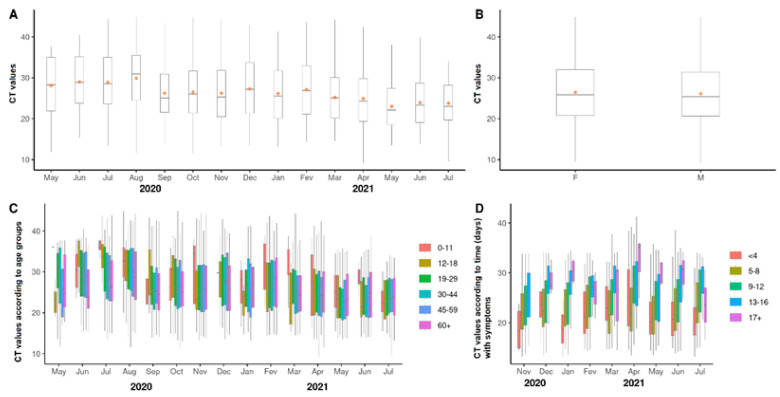
Viral loads according to cycle threshold (CT) values. (**A**) fluctuation of viral loads found in samples collected from the study population along the period of study. (**B**) comparison of viral loads according to gender. (**C**) viral loads according to age group for each month of the study. (**D**) viral loads according to time (days) with symptoms, from November 2020 to July 2021. Dots represent means. Horizontal bars represent medians.

The SARS-CoV-2 viral loads were inferred according to cycle thresholds (CT) found after RT-qPCR of the patient’s nasopharynx swabs. As shown in Figure 3A and Supplementary Table S5, we observed the fluctuation of CT values of SARS-CoV-2-positive samples during the study period. It was possible to observe increases and decreases in the mean CT values along the months. Interestingly, a tendency to decrease was observed in the last three months. The CT values were not significantly different according to gender, as shown in Figure 3B (*p* > 0.05). In addition, when CT values were compared along months according to age groups (Figure 3C), it was possible to observe that the age group of 60 and above years old presented lower CT values in June and July 2020 (*p* < 0.05) (see Supplementary Material S2 for statistical details). However, such a decrease was not seen in the other months of the study period. Moreover, patients with ages ranging from 0 to 18 years old presented significantly diminished or increased CT values in one or two months. However, we did not find a general tendency regarding viral loads according to age group. In contrast, the CT values were shown to be significantly lower in the beginning of the symptom period (*p* < 0.05), as shown in Figure 3D (see Supplementary Material S3, for statistical details). Collectively, these results indicate that the study population presented different viral loads in nasopharynx over time, as shown by CT values. However, the CT values did not differ relevantly according to age groups. In contrast, they were shown to be significantly reduced in samples collected at the early stage of COVID-19 symptoms, which indicates higher viral loads in this period.

### 3.4. Substitution of SARS-CoV-2 Lineages and Its Impact on Local Health

**Figure 4:**
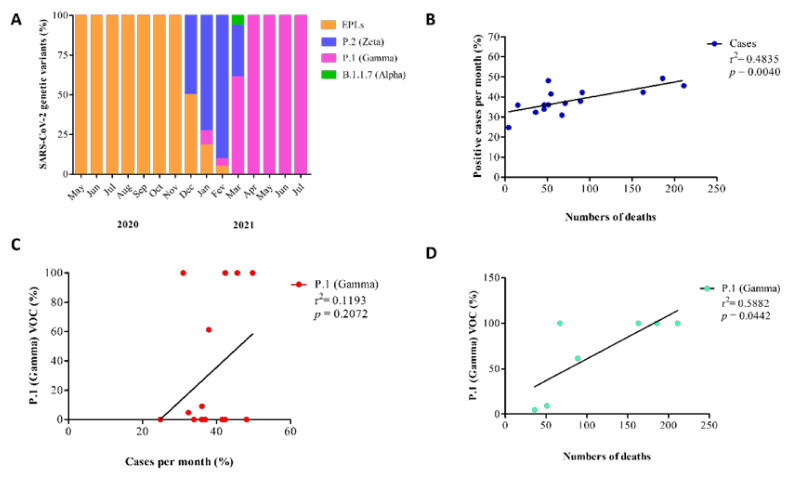
Substitution of SARS-CoV-2 lineages and its impact on local health. (**A**) Proportions of SARS-CoV-2 lineages found during the period of study. Viruses were classified based on genome sequencing and a specific RT-qPCR strategy capable of detecting specific mutations, as described in Section 2. EPLs, early pandemic lineages of SARS-CoV-2. (**B**) association between proportions of numbers of cases per month and numbers of deaths per month, as confirmed by linear regression analysis. (**C**) lack of association between proportions of viruses of the Gamma lineage found per month and proportions of cases per month (confirmed by linear regression analysis). (**D**) association between proportions of viruses of the Gamma lineage found per month and numbers of death per month (confirmed by linear regression analysis). Statistical significance was set as *p* ≤ 0.05.

As shown in Figure 4A, from May to November 2020, only early pandemic lineages (EPLs) of SARS-CoV-2 such as B1.1, B1.1.28, B1.1.33, and N9 were found. In December 2020, the P.2 (Zeta) variant of interest (VOI) lineage was found together with EPLs. In January and February 2021, the P.2 VOI was still detected in predominance over EPLs. However, the P.1 (Gamma) variant of concern (VOC) lineage was detected in low proportions in these months. In March 2021, the Gamma VOC dominated the scenario over Zeta and prevented the B.1.1.7 (Alpha) VOC lineage fixation. These results show that the Gamma VOC was introduced in the study area/population and dominated the scene. Moreover, from April to July 2021 the Gamma VOC completely dominated the scenario and was the only SARS-CoV-2 lineage detected in nasopharynx samples of the study population.

As shown in Figure 4B, the increase in the numbers of cases caused an increase in the numbers of deaths due to COVID-19. It is important to note that the peaks of COVID-19 cases coincided with those of deaths in this study. Thus, we carried out a linear regression analysis to see if the increase in the numbers of cases determined the numbers of deaths. To see if the increase in the numbers of cases was determined by the increase in the proportions of the Gamma VOC detection, we also conducted a regression analysis (Figure 4C), which revealed no association between these variables. However, when we analyzed the association of proportions of Gamma VOC detection and numbers of deaths, we saw that the increase in the proportions of Gamma VOC detection determined the increase in numbers of deaths (Figure 4D). These results collectively indicate that the substitution of early pandemic SARS-CoV-2 lineages by the Gamma VOC caused a significant impact on the health of the population studied, with a significant increase in the numbers of deaths due to COVID-19.

### 3.5. Viral Phylogeny

**Figure 5:**
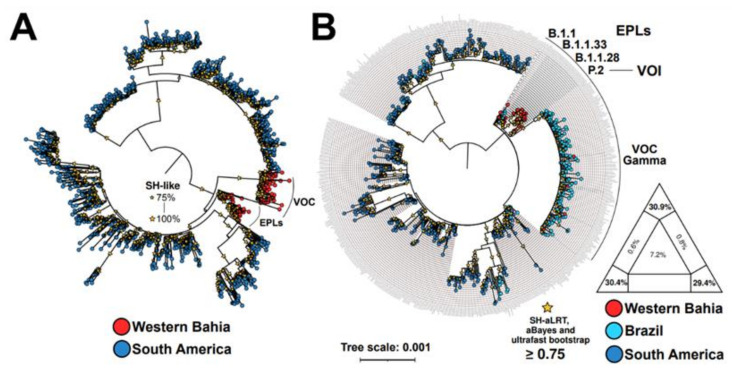
Maximum-likelihood midpoint rooted phylogenetic tree based on 1117 (A) and 603 (**B**) representative genome sequences of SARS-CoV-2. Nextstrain’s subsampled SARS-CoV-2 genomic data from South America (including Brazil) since pandemic started up to August 2022 containing unfiltered (**A**) and filtered (**B**) sequences from the study. The SARS-CoV-2 genomes from this study are identified by the red circles. Tips are colored according to sampling locations. Yellow stars assume Shimodaira–Hasegawa (SH-like) test (**A**) and SH-aLTR/aBayes/ultrafast bootstrap support (**B**) based on 1000 replicates. Only values equal or greater than 75% are shown. Likelihood mapping of the final sequences alignment showing low phylogenetic noise, as required for reliable phylogeny inference (**B**). Abbreviations: VOC and VOI, Variant(s) of Concern and Variant(s) of Interest, respectively. EPLs, Early Pandemic Lineages. In letter B, “Brazil” represents whole-genome of SARS-CoV-2 Gamma variant from all Brazilian States and the Federal District. Branch lengths are drawn in scale of nucleotide substitutions per site according to the bar scale. Colors and symbols used in the panels are defined according to the legend to the left and right of the figure.

To phylogenetically describe circulating viruses in Western Bahia during the study period we carried out maximum-likelihood analyses. As shown in Figure 5A, SARS-CoV-2 genomes of viruses that circulated in our study area during the study period were grouped separately from those found in other countries of South America. On the other hand, they grouped with viruses found in Brazil (Figure 5B). In addition, EPLs of SARS-CoV-2 were shown to have preceded VOC in the study as also shown in Figure 5B. This result confirms that EPLs of SARS-CoV-2 were replaced by VOCs, mainly the Gamma lineage, as shown in Figure 4A. Collectively, these results show that viruses found in the study area during the study period are phylogenetically related to Brazilian isolates and that EPLs of SARS-CoV-2 were replaced by the Gamma lineage.

## 4. Discussion

In this study, we aimed to have a picture of the epidemiological dynamics of COVID-19 in a population of almost 17,000 patients in a period of 15 months. We studied: (i) the fluctuations of COVID-19 cases and deaths due to COVID-19 during the period of study; (ii) the epidemiological behavior of COVID-19 according to patient gender, age, viral load and viral genetic variants; (iii) associations between these variables; and (iv) viral phylogeny.

We observed that the numbers of COVID-19 cases and deaths due to COVID-19 fluctuated over time. This was an expected result, once that fluctuations in the numbers of cases and deaths were also observed worldwide, as indicated by data from the World Health Organization [31]. So far, during the pandemic, several factors have had an impact on whether the numbers of COVID-19 cases and deaths are increasing or declining in specific locations. These factors include human behavior, infection prevention policies, viral genetic mutations, the number of people who are vulnerable because they have not developed some immunity, whether from natural infection or through vaccination, and the effectiveness of vaccines over time.

In our case, the national, state, and city government authorities adopted different policies over time. The first case of COVID-19 in Bahia was reported by the state health authority on 6 March 2020, nine days after the first case in Brazil [32]. This first case involved a history of travel to Europe. In addition, the first case in Western Bahia was reported on 21 March 2020, involving a history of travel to São Paulo city [32]. It is important to highlight that the Brazilian carnival took place in February 2020, just before the first cases in our study area. The carnival is a very popular festival, which promotes intense traveling movements and direct contact with people [33]. In addition, international travelers are very frequent during the festival. Surprisingly, none or negligible infection prevention policies regarding COVID-19 were adopted by all levels of government authorities at that time, even with the WHO declaring that COVID-19 constitutes a Public Health Emergency of International Concern (PHEIC) on 30 January 2020 [31]. Thus, the carnival seems to have contributed to bringing SARS-CoV-2 to Brazil, to the Bahia state, and to Western Bahia.

Following the introduction of COVID-19 in our study area, we observed a sharpened increase in the numbers of cases and deaths from June to August 2020. Although government and health authorities had launched prevention policies [34], the June celebrations, which are very popular in Northeast Brazilian states such as Bahia, seem to have impacted human behavior. Despite decrees imposing social and physical distancing, the lack of experience with the pandemic at that time seems to have impelled people to commemorate at private celebrations. After this event, the numbers of cases per day remained elevated in comparison with the first month of study, a probable result of the spread and multiplication of SARS-CoV-2 in the population. Such a situation was dramatically changed with the replacement of the early pandemic lineages (EPLs) of SARS-CoV-2 found in 2020 with the Gamma variant of concern (VOC) in 2021.

Although the vaccination has been initiated on January 2021 at the study area, the specific groups of elderly and health professionals were vaccinated first. This was imposed by the low availability of vaccines. The introduction of the Gamma lineage in the study area in early 2021 resulted in a total domination of the scenario by the new virus lineage. In addition to the replacement of the early pandemic lineages (EPLs) of SARS-CoV-2 in the study area, an increased mortality took place, most probably related to the increased pathogenicity of the Gamma lineage in comparison to the EPLs. In this context, it is important to highlight that the proportions of adults with ages ranging from 30 to 59 dying due to COVID-19 were increased at this period. This group was vaccinated with a delay in comparison to the elderly. 

In fact, all lineages of SARS-CoV-2 detected during the study produced more deaths in men than in women. It is well known that women have a better immune response than men. Generally, adult females mount stronger innate and adaptive immune responses than males. This results in the faster clearance of pathogens and greater vaccine efficacy in females than in males [35]. In addition, men tend to expose themselves more to risk [36]. These two factors seem to explain the results. On the other hand, the higher incidence of COVID-19 in people with age ranging from 30 to 44 does not have an easy explanation. We suppose that people with this age range are more economically and professionally active. They may have moved and contacted more people, exposing themselves more to infection.

Another interesting observation in our study was related to higher viral loads found in samples collected from patients in the early symptomatic period. This result by itself is in accordance with previous studies [37,38]. However, differences in viral loads were not observed according to the replacement of viral lineages. Interestingly, diminished viral loads are related to a worst outcome [38], but the more pathogenic Gamma lineage, which dominated the scenario in the five last months of study, was not detected with higher CT values (diminished viral load). It is important to highlight that a possible explanation for these results can be related not only to viral clearance, but also to the descending of infection from the nasopharynx to the lower respiratory tract, especially in severe cases.

Collectively, results presented in this study indicate that the numbers of COVID-19 cases and deaths due to COVID-19 fluctuated over time and that men were the most affected by deaths, as well as those of 60 or more years old. We also observed that individuals between 30 and 44 years old were the most affected by COVID-19 cases. In addition, the viral loads in the patient’s nasopharynx were higher in the early symptomatic period. Relevantly, we found that early pandemic SARS-CoV-2 lineages were replaced by the variant of concern (VOC) P.1 (Gamma) in the second half of the study period, which led to a significant increase in the number of deaths. Although the low number of samples subjected to genomic sequencing may generate limitations regarding the time of detection of viral lineages replacements, the main conclusions are supported by robust statistical analyses. In addition, genomic surveillance was complemented by genotyping using RT-qPCR. Thus, the results presented in this study are helpful for future formulations of efficient public policies of COVID-19 containment.

## Data Availability

We are submitting supporting data together with this manuscript. If necessary, additional data will be provided under request.

## Supplementary Materials

The following supporting information can be downloaded at: https://www.mdpi.com/article/10.3390/v14102314/s1. Material S1: Metadata; Material S2: Pairwise comparisons using *t* tests with pooled SD; Material S3: Pairwise comparisons using *t* tests with pooled SD; Table S1: Matrix with *p* values of comparisons (Bonferroni multiple comparison test after ANOVA) between each pair of months, regarding numbers of cases per day. Identification of months are shown in rows and columns. First, the number of the year is given and is followed by the number of the month, beginning at 1 (January) and ending at 12 (December); Table S2: Matrix with *p* values of comparisons (Bonferroni multiple comparison test after ANOVA) between each pair of months, regarding numbers of deaths per day. Identification of months are shown in rows and columns. First, the number of the year is given and is followed by the number of the month, beginning at 1 (January) and ending at 12 (December); Table S3: Absolute numbers of COVID-19 cases according to age group and month of study; Table S4: Absolute numbers of deaths due to COVID-19 according to age group and month of study; Table S5: Matrix with *p* values of comparisons (Bonferroni multiple comparison test after ANOVA) between each pair of months, regarding CT values. Identification of months are shown in rows and columns. First, the number of the year is given and is followed by the number of the month, beginning at 1 (January) and ending at 12 (December).
