# Supplementary material for: Impact of Early Pandemic SARS-CoV-2 Lineages Replacement with the Variant of Concern P.1 (Gamma) in Western Bahia, Brazil
Source: Viruses. 2022 Oct 21;14(10):2314. doi: 10.3390/v14102314 (PMC9611628; doi:10.3390/v14102314)
Supplement: Supplementary file 1 [file viruses-14-02314-s001.zip › Supplementary Tables S1-S5.pdf]

Supplementary Table S1 – Matrix with *p* values of comparisons (Bonferroni multiple comparison test after ANOVA) between each pair of months, regarding numbers of cases per day. Identification of months are shown in rows and columns. First, the number of the year is given and is followed by the number of the month, beginning at 1 (January) and ending at 12 (December).

POSITIVE CASES

Month vs month	2020_05	2020_06	2020_07	2020_08	2020_09	2020_10	2020_11	2020_12	2021_01	2021_02	2021_03	2021_04	2021_05	2021_06
2020_06	**0.00238**	-	-	-	-	-	-	-	-	-	-	-	-	-
2020_07	**5.6e-06**	**0.00362**	-	-	-	-	-	-	-	-	-	-	-	-
2020_08	**0.00053**	0.32490	**0.03592**	-	-	-	-	-	-	-	-	-	-	-
2020_09	**0.00394**	0.64203	**0.02148**	0.69679	-	-	-	-	-	-	-	-	-	-
2020_10	**0.00023**	0.03815	0.62740	0.17014	0.10969	-	-	-	-	-	-	-	-	-
2020_11	**0.02414**	0.92858	**0.01050**	0.40602	0.65644	0.05961	-	-	-	-	-	-	-	-
2020_12	0.07625	0.26251	**0.00066**	0.06667	0.18390	**0.00897**	0.44002	-	-	-	-	-	-	-
2021_01	0.11528	0.92468	**0.04333**	0.52679	0.71776	0.12121	0.97675	0.59767	-	-	-	-	-	-
2021_02	0.06196	0.47402	**0.00215**	0.15215	0.32179	**0.01956**	0.62181	0.79504	0.73442	-	-	-	-	-
2021_03	0.43426	0.09808	0.00030	**0.02605**	0.07313	**0.00371**	0.19468	0.48104	0.33406	0.37915	-	-	-	-
2021_04	**4.7e-05**	**0.02199**	0.49858	0.14968	0.09153	0.90156	**0.04636**	**0.00420**	0.11886	**0.01174**	**0.00177**	-	-	-
2021_05	0.00099	0.24736	0.09659	0.75420	0.52406	0.30179	0.30830	**0.05890**	0.42022	0.12116	**0.02351**	0.30288	-	-
2021_06	**0.03713**	0.86421	0.03943	0.64284	0.87568	0.13283	0.83257	0.38477	0.84310	0.52456	0.18545	0.12564	0.50488	-
2021_07	0.14099	0.09566	**0.00017**	**0.02029**	0.07933	**0.00350**	0.25074	0.67508	0.43355	0.51757	0.69872	**0.00120**	**0.02210**	0.24048

Supplementary Table S2 – Matrix with *p* values of comparisons (Bonferroni multiple comparison test after ANOVA) between each pair of months, regarding numbers of deaths per day. Identification of months are shown in rows and columns. First, the number of the year is given and is followed by the number of the month, beginning at 1 (January) and ending at 12 (December).

DEATHS

Month vs month	2020_05	2020_06	2020_07	2020_08	2020_09	2020_10	2020_11	2020_12	2021_01	2021_02	2021_03	2021_04	2021_05	2021_06
2020_06	**0.03793**	-	-	-	-	-	-	-	-	-	-	-	-	-
2020_07	**0.00022**	**0.00527**	-	-	-	-	-	-	-	-	-	-	-	-
2020_08	**1.3e-09**	**2.9e-07**	0.10057	-	-	-	-	-	-	-	-	-	-	-
2020_09	**2.7e-06**	**0.00026**	0.68315	0.16912	-	-	-	-	-	-	-	-	-	-
2020_10	**1.2e-06**	**0.00946**	0.18183	**0.00015**	**0.03847**	-	-	-	-	-	-	-	-	-
2020_11	**0.00042**	**0.07132**	0.12050	**0.00015**	**0.02524**	0.63663	-	-	-	-	-	-	-	-
2020_12	**0.00027**	**0.06459**	0.11672	**0.00012**	**0.02337**	0.62768	1.00000	-	-	-	-	-	-	-
2021_01	**0.00011**	**0.04092**	0.14641	**0.00019**	**0.03211**	0.76991	0.86369	0.86079	-	-	-	-	-	-
2021_02	**0.00505**	**0.29109**	**0.04078**	**1.8e-05**	**0.00545**	0.20717	0.48486	0.47601	0.37455	-	-	-	-	-
2021_03	**4.8e-08**	**2.9e-06**	0.10523	**0.91489**	**0.17481**	**0.00063**	**0.00047**	**0.00041**	**0.00060**	**7.7e-05**	-	-	-	-
2021_04	**8.7e-13**	**2.0e-12**	**9.0e-09**	**1.6e-07**	**7.7e-09**	**4.9e-11**	**2.1e-11**	**2.1e-11**	**2.8e-11**	**6.4e-12**	**4.5e-07**	-	-	-
2021_05	**4.3e-12**	**1.1e-11**	**6.9e-09**	**1.3e-07**	**8.9e-09**	**1.9e-10**	**8.8e-11**	**8.9e-11**	**1.1e-10**	**3.2e-11**	**2.5e-07**	0.37667	-	-
2021_06	**8.6e-12**	**3.6e-11**	**6.1e-07**	**1.4e-05**	**5.8e-07**	**1.4e-09**	**7.3e-10**	**6.9e-10**	**9.3e-10**	**1.8e-10**	**3.7e-05**	0.20137	**0.04330**	-
2021_07	**2.6e-06**	**0.00019**	0.60069	**0.21706**	**0.89712**	**0.02798**	**0.01850**	**0.01706**	**0.02356**	**0.00391**	0.21905	**1.2e-08**	**1.2e-08**	**9.0e-07**

Supplementary Table S3. Absolute numbers of COVID-19 cases according to age group and month of study.

	2020								2021						
Age group	May	Jun	Jul	Aug	Sep	Oct	Nov	Dec	Jan	Feb	Mar	Apr	May	Jun	Jul
0-11	0	4	2	12	9	13	2	1	2	5	3	4	21	14	15
12-18	6	5	11	18	10	18	16	10	3	18	11	43	47	38	36
19-29	20	76	97	105	88	86	102	48	44	64	39	122	151	134	73
30-44	41	102	202	191	135	181	137	90	50	124	106	225	284	238	138
45-59	12	60	102	94	105	109	89	46	51	67	69	160	234	183	71
60+	9	22	69	120	79	105	85	59	57	70	81	152	129	95	55

Supplementary Table S4. Absolute numbers of deaths due to COVID-19 according to age group and month of study.

	2020								2021						
Age group	May	Jun	Jul	Aug	Sep	Oct	Nov	Dec	Jan	Feb	Mar	Apr	May	Jun	Jul
0-11	0	0	0	0	1	0	0	0	0	0	0	0	2	0	0
12-18	0	0	0	0	0	0	0	0	0	0	1	0	0	1	1
19-29	0	0	1	1	2	0	0	0	1	0	3	4	2	5	0
30-44	1	2	7	8	6	4	3	5	3	4	11	19	35	37	14
45-59	2	3	14	11	18	6	5	5	7	3	16	48	75	55	19
60+	0	10	32	67	44	41	38	36	40	29	58	115	96	64	33

Supplementary Table S5 – Matrix with *p* values of comparisons (Bonferroni multiple comparison test after ANOVA) between each pair of months, regarding CT values. Identification of months are shown in rows and columns. First, the number of the year is given and is followed by the number of the month, beginning at 1 (January) and ending at 12 (December).

Month vs month	2020_05	2020_06	2020_07	2020_08	2020_09	2020_10	2020_11	2020_12	2021_01	2021_02	2021_03	2021_04	2021_05	2021_06
2020_06	**0,00238**													
2020_07	**5,60E-06**	**0,00362**												
2020_08	**0,00053**	0,3249	**0,03592**											
2020_09	**0,00394**	0,64203	**0,02148**	0,69679										
2020_10	**0,00023**	0,03815	0,6274	0,17014	0,10969									
2020_11	0,02414	0,92858	**0,0105**	0,40602	0,65644	0,05961								
2020_12	0,07625	0,26251	**0,00066**	0,06667	0,1839	**0,00897**	0,44002							
2021_01	0,11528	0,92468	**0,04333**	0,52679	0,71776	0,12121	0,97675	0,59767						
2021_02	0,06196	0,47402	**0,00215**	0,15215	0,32179	**0,01956**	0,62181	0,79504	0,73442					
2021_03	0,43426	0,09808	**0,0003**	**0,02605**	0,07313	**0,00371**	0,19468	0,48104	0,33406	0,37915				
2021_04	**4,70E-05**	**0,02199**	0,49858	0,14968	0,09153	0,90156	**0,04636**	**0,0042**	0,11886	**0,01174**	**0,00177**			
2021_05	**0,00099**	0,24736	0,09659	0,7542	0,52406	0,30179	0,3083	0,0589	0,42022	0,12116	**0,02351**	0,30288		
2021_06	0,03713	0,86421	**0,03943**	0,64284	0,87568	0,13283	0,83257	0,38477	0,8431	0,52456	0,18545	0,12564	0,50488	
2021_07	0,14099	0,09566	**0,00017**	**0,02029**	0,07933	**0,0035**	0,25074	0,67508	0,43355	0,51757	0,69872	**0,0012**	**0,0221**	0,24048
